# Psychiatric disorders in children and adolescents in a middle-income Latin American country

**DOI:** 10.1186/s12888-020-02512-4

**Published:** 2020-03-05

**Authors:** Alejandra Caqueo-Urízar, Jerome Flores, Carolang Escobar, Alfonso Urzúa, Matías Irarrázaval

**Affiliations:** 1 Instituto de Alta Investigación, Universidad de Tarapacá & Centro de Justicia Educacional, 1520 Antofagasta, Arica Chile; 2 Escuela de Psicología y Filosofía, Universidad de Tarapacá, Centro de Justicia Educacional, CJE, Arica, Chile; 3 Escuela de Psicología, Universidad Católica del Norte, Antofagasta, Chile; 4 Departamento de Psiquiatría, Faculta de Medicina, Hospital Clínico, Universidad de Chile, Santiago, Chile

**Keywords:** Psychiatric disorders, Infantile-juvenile population, Latin America, Early intervention

## Abstract

**Background:**

Child and adolescent mental health has scarcely been studied in developing countries, though it is an important aspect of health. Mental health problems in youth often continue into adulthood if not diagnosed or treated in time.

**Methods:**

The *Sistema de Evaluación de Niños y Adolescentes (SENA)* [Child and Adolescent Evaluation System] was used to evaluate mental health indicators in a sample of students in Northern Chile. Two age-appropriate versions of the assessment were applied to a total sample of 5043 students, which included an elementary education sample of 1953 schoolchildren from fourth grade through sixth grade (ages 8 to 13 years), and a Secondary School sample of 3090 schoolchildren from seventh grade (the last year of elementary school) through the last year of Secondary school (senior high school) (ages 12 to 19 years). For each group, the version of the assessment used was determined by the students’ grade level. Both samples included municipal, government-subsidized, and private schools.

**Results:**

In this student population, depression, anxiety, and behavioral disorders were the main mental health problems identified, and indicators revealed a progressive increase in cases over the years, coinciding with the global epidemiological scenario. Males showed a greater presence of externalizing behaviors related to mental health problems associated with aggression and defiant behavior. However, females showed the highest number of mental health issues overall, especially regarding problems related to internalization. There are significant differences between school types.

**Conclusions:**

Our sample population analysis indicates that early intervention is necessary for the diagnosis and treatment of youth, with the goal of reducing the probability that psychiatric disorders will be prolonged, evolving, and worsening in adulthood.

## Background

Mental health problems during infancy, childhood, or adolescence affect the processes of adaptation and functionality in adulthood so mental health issues in the youth population should be considered a priority for the management of public health [1–3].

The prevalence of mental health problems in young children is estimated to be between 17 and 20% globally, with the first symptomatic manifestations observed before age 14 on average. In addition, it has been found that the highest prevalence rates are in developing countries [4, 5].

However, intervention is complicated, as findings of high comorbidity rates of mental health disorders suggest that the complexity of diagnosis and treatment is a major challenge. Between 24 and 29% of the mental health problems usually diagnosed in young children, such as depression, anxiety, and behavioral disorders, often contain characteristics that are also present in other disorders [6, 7]. For example, depression in this age group is often comorbid with anxiety and/or behavioral disorders. It also often involves somatic symptoms, such as gastrointestinal discomfort, headaches, muscle aches, excessive tiredness, and sleep or appetite disturbances, together with other internalizing symptoms of an affective and emotional states associated with hypersensitivity, irritability, aggressiveness, impulsivity, social withdrawal, demotivation, intolerance to frustration, and low self-esteem. In addition to symptomatic expression at the cognitive level, depression in youth usually includes difficulties in paying attention, concentrating, understanding, and remembering, thereby negatively affecting learning processes. It should be noted that in adolescence, depression may also be accompanied by problematic substance use, eating disorders, anti-social behavior, or suicidal ideation [8, 9].

The prevalence of depressive disorders in children and adolescents is estimated to be increasing, and the average age at which the first signs or symptoms appear is decreasing. Globally, the prevalence ranges from 2 to 5% in children and 4 to 8% in adolescents. Learning and emotional difficulties are also prevalent, and these have been shown to contribute to the development of depressive symptoms [10–12]. Thus, depression may have the most comorbidity with other mental health problems.

On the other hand, the prevalence of anxiety disorders in children and adolescents significantly surpasses that of other mental health problems usually diagnosed in this population (4 to 32% worldwide), not including those cases in which anxiety manifests itself as a symptom of other clinical conditions. Anxiety also shows comorbidity with social phobia and obsessive-compulsive disorders, and it impacts not only the affective domain but also cognitive functions.

Behavioral disorders make up another category of mental health problems that can occur in childhood and adolescence. Owing to their overt nature, behavioral disorders mainly affect social development such as in school, social, and family contexts; in addition, they have a direct correlation with adaptation disorders in adulthood. Behavioral disorders have a worldwide prevalence ranging from 5 to 7% in children and between 2 and 4% in adolescents [13]. Diagnoses of this type of disorder are higher in males, with more cases of attention-deficit/hyperactivity disorder (ADHD), which significantly affects socialization and learning processes reported in childhood, and more cases of oppositional defiant disorders (ODD) and dissocial personality disorders (DPD, or anti-social personality disorders) reported in adolescence [13]. ODD and DPD share in common characteristics associated with opposition to norms, defiant behavior vis-á-vis authority figures, irritability, and recurring emotional states of anger, along with difficulties in managing these emotions and behaviors. Impulsivity and aggression are also observed with ODD and DPD [14].

In Chile, the prevalence of mental health problems in children is 38.2%, with behavioral disorders associated with attentional deficit with hyperactivity being the most common, followed by anxiety disorders (of which separation anxiety is the most prevalent), and finally, mood disorders, of which major depression has the highest prevalence [15–18].

Several risk factors related to the mental health of young children have been identified, including low socioeconomic level, family dysfunction, physical or psychological abuse, traumatic experiences (accidents, natural disasters, wars, etc.), and insufficient stimulation. Conversely, sensitive upbringing with responsible parenting, including stimulation for the autonomous development of children and educational opportunities for development, has been shown to be a protective factor against mental health disorders [19].

It has been observed that school plans and programs aimed at safeguarding the well-being of students have managed to detect, in a timely manner, problems usually related to depression, anxiety, and behavioral disorders, in addition to mitigating the negative impact of the insufficient investment of public resources in mental health and the low use of available services by the general population. A low rate of service use has been identified as a common problem in several countries, and may be caused by the fear of social stigma associated with a diagnosis, lack of information, or the uncertainty of parents or caregivers regarding whether the changes in behavior or mood observed in their children require treatment [20].

These statistics clearly suggest that the need to detect symptoms of mental health problems and intervene in a timely manner presents a relevant challenge for educational systems, as strategies that facilitate teaching and learning processes also safeguard the psychological well-being of students [20]. In order to meet this challenge and accurately assess psychopathologies that affect optimal growth and development in children and adolescents, the use of valid and reliable instruments for comprehensive psychological assessment is key.

It is essential to study the frequency of mental disorders in children and adolescents in scarcely studied contexts, such as in Latin America and especially in Chile, which is currently experiencing a severe social and economic crisis marked by significant social inequality and classism.

One such reliable instrument of assessment available is the *Sistema de Evaluación de Niños y Adolescentes (SENA)* [Child and Adolescent Evaluation System] [21], which facilitates the estimation of mental health problems common in the youth population as well as providing an assessment of personal resources and risk factors related to modes of interaction in family, social, and school contexts. The *SENA* also evaluates for specific problems, such as substance abuse, eating disorders, learning problems, developmental delay, and unusual or disruptive behavior.

The aim of this study was to conduct a descriptive analysis of mental health indicators in a sample of Elementary and Secondary School students in Northern Chile using the *SENA*.

## Methods

This was a non-experimental study since variables were not manipulated. The design was cross-sectional correlational, because all measured variables were collected at the same time [21].

### Participants

The total sample included 5043 students. Two samples were assessed, and different versions of the *SENA* were applied according to the students’ grade levels. Both samples included municipal, government-subsidized and private schools.

The Elementary School sample was 1953 students between the 4th and 6th grades. Of the participants, 48.6% were male and 51.4% were female. The range of age was from 8 to 19 years old. The mean age was 10.3 years and the standard deviation was 0.9 years. Additionally, 40.8% attended municipal schools, 53.7% attended establishments subsidized by the government, and 5.5% attended private schools. Regarding participants’ nationalities, the population sample was 88.9% Chilean, 5.3% Bolivian, 3.4% Peruvian, 0.5% Colombian, 0.5% Venezuelan, 0.3% Argentinean, 0.1% Ecuadorian, and 0.9% did not specify their nationality.

The Secondary School sample consisted of 3090 students between 7th grade and the fourth year of High School. Of the participants, 48% were male and 52% were female. The mean age was 14.6 years and the standard deviation was 1.8 years. Additionally, 42% attended municipal establishments, 53% attended establishments subsidized by the government, and 4.7% attended private schools. Regarding nationality, the population sample was 93% Chilean, 3.1% Bolivian, 2.3% Peruvian, 0.6% Colombian, 0.2% Venezuelan, 0.2% Ecuadorian, and 0.3% did not specify their nationality.

### Instrument

The *Sistema de Evaluación de Niños y Adolescentes* (*SENA*) [22] was developed by specialists in psychopathology and psychological evaluation. Its purpose is to help in the detection of a wide range of emotional and behavioral problems in individuals between 3 to 18 years of age. It is worth noting that it has been constructed and validated entirely in the Spanish language. Among the problems it assesses for are the following:

*Internalized problems:* depression, anxiety, social anxiety, somatic complaints, post-traumatic symptomatology, and obsession-compulsion.

*Externalized problems:* attention problems, hyperactivity-impulsivity, anger control problems, aggression, defiant behavior, and anti-social behavior.

*Contextual problems:* problems with family, problems with school, and problems with peers.

*Specific problems:* substance use, eating behavior problems, schizotype, or unusual behavior.

Other areas we assessed using the *SENA* included problems of *emotional regulation* and *search for sensations*. These are all associated with the onset or maintenance of more serious disorders.

The elementary school version (8–12 years old) has 16 scales, 11 of which measure mental health, 3 contextual problems and 2 personal resources. The Secondary School version has 23 scales, 17 of which measure mental health, 3 contextual problems and 3 personal resources.

Each of the dimensions varies between 3 and 14 items, with an average of 8 items per scale. Some examples of the content are: “I feel that no one cares what I do” (Depression), “I get anxious or overwhelmed by my problems” (Anxiety), “I get nervous when there are many people around” (Social Anxiety). “I get unpleasant images of things that have happened to me” (Post-Traumatic Stress), “I get attention in class because I can’t stop moving” (Hyperactivity-Impulsivity), “When I go out to have fun I take drugs” (Substance abuse), “I have problems at home” (Family problems). “I find it exciting to do dangerous things” (sensation seeking). “I have trouble controlling my emotions (emotional regulation), I threaten others to get what I want (aggression).

Additionally, the *SENA* evaluates for the presence of certain personal resources, such as *self-esteem*, *integration, social competence,* and *problem awareness*. These may be considered protective and/or supportive factors of clinical intervention. Control scales include *positive print, negative print, and inconsistency.*

The *SENA* employs a Likert scale that ranges from 1 to 5 in the answer options for each item, from “never or almost never” (1) to “always or almost always” (5). The total of each dimension is the average of the total of the answers that constitute it, ranging from 1 to 5.

Recently, Sánchez-Sánchez, Fernández-Pinto, Santamaría, Carrasco, and del Barrio (2016) [22] found that the reliability of the subscales is greater than .7 in Spain. These results are consistent with the reliability of the dimensions in this study.

This research used the self-report versions of *SENA* for both Elementary (8 to 12 years) and Secondary School (12 to 18 years). The Secondary School version has several dimensions not present in the elementary school version, namely *obsessive-compulsive, antisocial behavior, schizotype, substance use, sensation seeking, eating behavior problems,* and *problem awareness.*

### Procedure and ethical considerations

We applied the following four steps to complete the study:

1. We gained the approval of the Ethics Committee of the University of Tarapacá. This study is part of a larger Educational Justice Centre project.
2. Forty-two educational establishments in the city of Arica were invited to participate in the study, with 69% agreeing to participate, for a total of 29 establishments.
3. Consent was requested from the parents of all participating students, after the purpose and scope of the study was explained to them; then, the assent of the students themselves was requested.
4. Assessments were conducted in groups at each grade level. Along with the grade-level teacher, at least two trained interviewers were present at each assessment to answer questions about the meaning of the items or how to respond. The duration of each group’s assessment was approximately 45 min.

### Data analysis

**Fig. 1:**
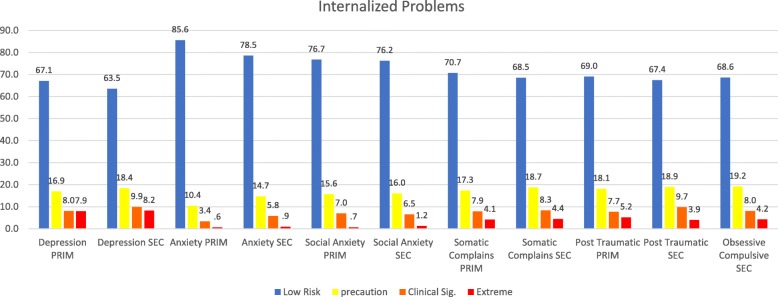
Percentage of levels in dimensions of internalized problems in a sample of school students in Northern Chile, using standard scores. Note1: PRIM is Elementary School and SEC is Secondary School. Note 2: Obsessive-compulsive exists only in Secondary School version

The demographics of each sample, followed by basic statistics on the dimensions, as well as the correlations between them, are presented in Tables 1, 2, and 3. The graphs for the internalized and externalized problems, contextual problems, vulnerability, others problems and personal resources of both samples are shown in Figs. 1 through 6. The values for both age groups were classified according to the criteria in the *SENA* manual. In the scales depicting problems and vulnerability, it is important to differentiate the highest scores, and we considered a standard score > 60 to fall within the “caution” zone, a score > 70 to fall within the “clinically significant” zone, and a score > 80 to fall within the “extreme” zone. In the scales depicting personal resources, it is important to differentiate the lowest scores, and it is considered that a standard score < 39 falls within the “precautionary” zone, < 29 falls within the “clinically significant” zone, and < 19 falls within the “extreme” zone.

**Table 1:** Means, Standard Deviations and possible range in global indices of the Elementary School SENA in a sample of 1953 Chilean students from 4th and *6th grades*

	*M*	*SD*
Global index	52,5	8,43
Index of Emotional problems	53,7	9,5
Index of Behavioral problems	51,0	10,1
Index of Executive functions	52,3	9,3
Index of Contextual problems	52,7	10,3
Index of Personal resources	43,1	10,9

Score < 39 is considered low, between 40 and 59 is considered medium, and > 60 is considered high

**Table 2:** Means, Standard Deviations and Possible Range in Global Indices of the SENA in a sample of 3090 Chilean Secondary School Students from 7th grade and the fourth year of High School

	*M*	*SD*
Global index	52,3	7,9
Index of Emotional problems	54,2	9,8
Index of Behavioral problems	50,6	9,0
Index of Executive functions	51,7	8,8
Index of Contextual problems	51,4	8,6
Index of Personal resources	42,0	10,8

Score < 39 is considered low, between 40 and 59 is considered medium, and > 60 is considered high

**Table 3:** MANOVA analysis of estimated marginal means and significance for Sex and Educational Level in a sample of Chilean youth

VARIABLE	Sex (S)		Educational Level (EL)		Sex (S)^a^	EL^b^	S x EL^c^
	Woman	Men	Elementary	Secondary			
	*M*	*M*	*M*	*M*	*p*	*p*	*p*
Depression	2,1	2,0	2,0	2,1	,000***	,000***	,000***
Anxiety	2,5	2,3	2,2	2,5	,000***	,000***	,000***
Social Anxiety	2,5	2,3	2,4	2,4	,000***	0,081	,043*
Somatic Complains	2,3	2,1	2,2	2,3	,000***	,000***	,000***
Post Traumatic	2,2	2,1	2,2	2,1	,000***	,000***	,000***
Attention Problems	2,3	2,3	2,3	2,4	0,109	,000***	,005**
Hiperactivity	1,9	2,1	1,9	2,1	,000***	,000***	,000***
Anger Control Problems	2,0	2,0	1,9	2,1	0,528	,000***	,000***
Aggressiveness	1,3	1,4	1,3	1,4	,000***	,000***	0,444
Defiant Behaviour	1,5	1,6	1,5	1,6	,005**	,000***	,015*
Family Problems	1,7	1,7	1,6	1,8	0,455	,000***	,000***
School Problems	1,7	1,9	1,7	1,9	,000***	,000***	0,051
Peer Problems	1,3	1,5	1,5	1,3	,000***	,000***	0,855
Emotional Regulation Problems	2,5	2,2	2,3	2,4	,000***	,000***	,000***
Self-esteem	3,7	3,9	3,9	3,7	,000***	,000***	,000***
Integration and social competence	3,5	3.43	3,5	3,4	,002**	,000***	0,172

^a^Main effect of sex, ^b^Main effect of Educational Level, ^c^Interaction between Sex and Educational Level

*p<,05 **p<,01 ***p<,000

**Fig. 2:**
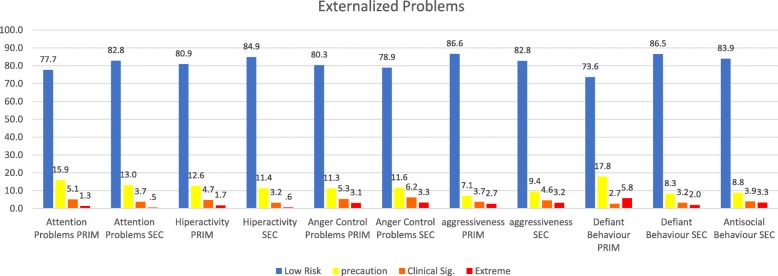
Percentage of levels in dimensions of externalized problems in a sample of school students in Northern Chile, using standard scores. Note 1: PRIM is Elementary School and SEC is Secondary School. Note 2: Antisocial Behaviour exist only in Secondary School version

**Fig. 3:**
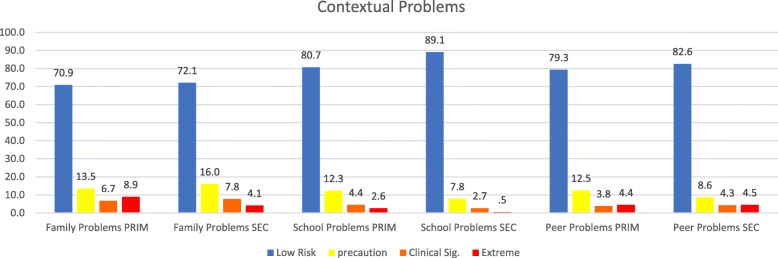
Percentage of levels in dimensions of contextual problems in a sample of school students in Northern Chile, using standard scores. Note 1: PRIM is Elementary School and SEC is Secondary School

**Fig. 4:**
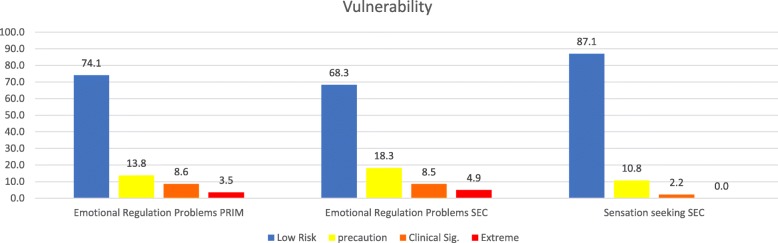
Percentage of levels in dimensions of Vulnerability in a sample of school students in Northern Chile, using standard scores. Note 1: PRIM is Elementary school and SEC is Secondary School. Note 2: Sensation seeking only exist in Secondary School version

**Fig. 5:**
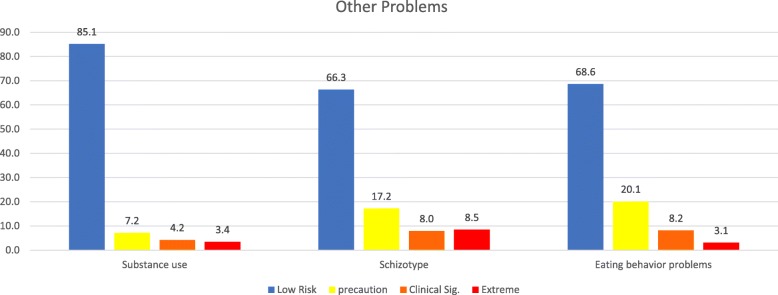
Percentage of levels in dimensions of Other problems in a sample of school students in Northern Chile, using standard scores. Note 1: These three dimensions only exist in Secondary School version

**Fig. 6:**
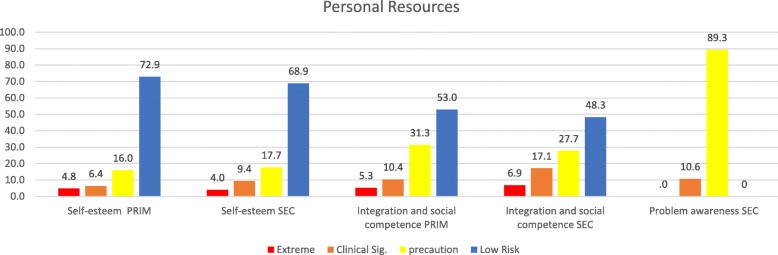
Percentage of levels in dimensions of Personal Resources in a sample of school students in Northern Chile, using standard scores. Note 1: PRIM is Elementary School and SEC is Secondary School. Note 2: Problem awareness only exist in Secondary School version

Finally, a multivariate analysis of variance (MANOVA) is presented, which includes as independent variables sex and educational level, and as dependent variables all scales of problems and vulnerability, as well as personal resources. SPSS version 22 was used for the statistical analyses.

## Results

The means, standard deviations, and possible range of the global indices of the *SENA* results in the Elementary and Secondary School sample are shown in Tables 1 and 2.

Percentages for internalized problems and externalized problems in the Elementary School sample are shown in Fig. 1 and Fig. 2, respectively. This data shows that percentages were higher in the precautionary area, the clinical significance zone, and the extreme area for internalized problems.

Figure 3 shows the percentage of levels in dimensions of contextual problems in the total sample. As is shown in these figures, percentages were higher in the precautionary area, the clinical significance zone, and the extreme area for internalized problems.

Table 3 summarizes the marginal means by sex (male and female) and educational level (Elementary or Secondary School), as well as the significant results for MANOVA. The assumption of multivariate homoscedasticity in the BOX test was not met; however, this would not significantly impact the analysis, especially if the groups included more than 30 participants [23, 24]. This suggests that it is appropriate to select Pillai’s Trace as the most robust test statistic.

A significant effect of sex was found, Pillai’s Trace = .131(16,5024) = 47.239, *p* = .000, η2 = .131. We found a significant effect of educational level, Pillai trace = .167(16,5024) = 63,151, *p* = .000, *η2* = .167. The effect of the interaction of sex and educational level was also significant, Pillai trace .027(16,5024) = 8746, *p* = .000, *η2* = .027.

Six of the fourteen scales depicting problems and vulnerability were significantly higher in females, five were higher in males, and three did not show any difference.

Depression, anxiety, social anxiety, somatic complaints, post-traumatic symptomatology, and emotional regulation problems were greater in females, while hyperactivity-impulsivity, aggressiveness, defiant behavior, problems with school and problems with peers were greater in males. Attention problems, anger management problems, and family problems were not significantly different between males and females. Females had significantly lower self-esteem than males, and males had less integration and social competence than females.

Secondary school students presented significantly higher values in eleven of the fourteen scales for problems and vulnerability. Specifically, they scored higher for depression, anxiety, somatic complaints, attention problems, hyperactivity, anger management problems, aggressiveness, defiant behavior, family problems, school problems, and emotional regulation problems. Meanwhile, post-traumatic symptomatology and peer problems were greater in elementary school students. The only item that did not differ between the two groups was social anxiety. In terms of personal resources, self-esteem and integration and social competence were lower in Secondary School students.

An interaction effect was found in all variables except aggressiveness, problems with school, problems with peers, and social integration and competence.

Figure 4 shows the Vulnerability dimension in both samples (Emotional Regulation Problems and Sensation Seeking (Only in Secondary School).

Figure 5 shows the results of Other Problems Dimension with three dimensions that only exist in Secondary School version (Substance use, Schizotype and Eating behaviors problems).

Figure 6 shows the percentage of levels in dimension of Personal Resources in both samples, using standard scores (Self-steem, Integration and Social Competence and Problem awareness that only exist in Secondary School version).

Finally, the differences in relation to school types were analyzed separately, because students from private schools represent less than 10% of the sample. In order to conduct an appropriate analysis for this variable, random subsamples of municipal (*n* = 260) and subsidized (*n* = 258) schools respectively, equivalent to private schools (*n* = 254), were selected. In this new sample of 772, the possible differences between the educational establishments were analyzed, obtaining only two significant variables: problems with the family and social integration and competence.

A significant effect of educational dependence was found, Pillai’s trace = .124 (32, 1492) = 3073, *p* = .000, η2 = .124. This was significant only in problems with family (*p <* .01), and in social integration and competence (*p <* .001). Post-hoc analyses showed that municipal schools differed significantly from subsidized (*p <* .01) and private schools *(p <* .01), but not between subsidized and private. Students from municipal schools present greater problems with the family (*M* = 1.77) than those subsidized (*M* = 1.62) and private (*M* = 1.59). Regarding integration and social competence, post-hoc analyses showed that students from private schools (*M* = 3.73) obtained greater integration and social competence than municipal (*M* = 3.34) and subsidized (*M* = 3.46) students. The latter two schools were not significantly different from each other.

A significant effect of interaction between school types and educational level was also found, Pillai’s trace = .066 (32, 1492) = 1.594, *p* = .019, η2 = .066. Specifically for depression (*p <*,01), social anxiety (*p <*,05), peer problems (p <,05), self-esteem (*p <*,01) and social integration and competence (*p <*,05).

Private school students have the lowest scores for depression in Elementary School (*M* = 1.8), but the highest scores in Secondary School (*M* = 2.2).

Social anxiety of students in subsidized schools decreases from Elementary (*M* = 2.6) to Secondary School (*M* = 2.3). In municipal schools, it remained the same from Elementary (*M* = 2.4) to Secondary School (*M* = 2.4) and in private schools it increased slightly from Elementary (*M* = 2.3) to Secondary School (*M* = 2.4).

Students in municipal schools decrease their problems with their classmates from Elementary (*M* = 1.5) to Secondary School (*M* = 1.3), while those in subsidized and private schools increase them.

Private school students have the highest self-esteem scores in Elementary School (*M* = 4.2), but the lowest in Secondary School (*M* = 3.6).

With respect to social integration and competence, the most important change is observed in private schools, which decrease from Elementary (*M* = 4) to Secondary School (*M* = 3.6), while in municipal schools it remains from Elementary (*M* = 3.3) to Secondary School (*M* = 3.3), and in subsidized schools it decreases slightly from Elementary (*M* = 3.5) to Secondary School (*M* = 3.4).

## Discussion

The aim of this study was to describe the mental health of students in an under-developed nation using indicators measured by the *SENA* in a sample of Elementary and Secondary School students in Northern Chile.

The results showed that in this youth population, depressive, anxious, and behavioral disorders continue to be the main mental health problems in Elementary and Secondary School students. Furthermore, indicators revealed progressive increases in prevalence during recent years, a finding that coincides with the world epidemiological scenario [25]. Pharmacological and psychotherapeutic treatments are called for, together with educational interventions and other services that not only ensure coverage and quality, but also a timely diagnosis, considering the evolutionary characteristics of development existing between childhood and adolescence [26–28].

Additionally, males showed a higher prevalence of externalizing mental health problems related to aggression and defiant behavior; however, it was females who had the highest rates of mental health problems overall, especially internalized problems. For females, low self-esteem was the main depressive symptom, parallel to somatic complaints, anxiety, and social anxiety. It has been shown that the closer a person gets to adulthood, the greater the probability is that these problems will increase over time. This finding corresponds to results from other studies indicating that anxiety and mood disorders tend to worsen and extend into adulthood to a greater degree than behavioral disorders [29, 30], with different incidence rates according to gender. The highest frequency of psychopathologies of an emotional or affective nature is found in the female population [31].

Among the areas of vulnerability that predispose students to present more severe mental health problems, greater difficulties were found in regulating emotions by attending to, perceiving, and clearly expressing their own emotional states, as well as managing the intensity and duration in an appropriate way, and moderating impulses or frustrations, especially in situations of conflict. This is a finding which, according to other studies, is usually related to anxiety disorders, in which the existence of recurrent and intense fears of the negative evaluation of others makes probable the appearance of insecurities that affect cognitive and especially emotional processes. This behavior can lead to students becoming increasingly evasive and resistant to contact with others. Since the emotionally destabilizing situations are all those where they are exposed to judgement or criticism [32–36].

Another important finding related to vulnerability was the search for sensation in the adolescent population, whereby the need to try varied, complex and intense experiences, the desire to take risks, and the susceptibility to boredom increased the probability of exposing oneself to dangers. The search for sensation is characteristic of adolescence, together with susceptibility to influence, fantasy of immunity or invulnerability, and the search for or reaffirmation of identity, autonomy, and group integration where the adoption of risk behaviors is expected [37]. Some studies suggest a relationship between sensation-seeking and exposure to risks, with the existence of behavioral disorders linked to hyperactivity as well as defiant and anti-social behavior [38, 39].

We found that the risk factors that do not allow students to face situations perceived as problematic or threatening are due to the lack of social competence, that is, a lack of ability to establish and maintain positive relationships with classmates, and to communicate and behave in an assertive manner while respecting individual or group differences. The degree to which a student has difficulty resolving interpersonal conflicts is based on their awareness and level of self-esteem, as a negative evaluation of self-concept contributes to a reduced capacity for conflict resolution. These findings corresponded with those of other studies in which the excessive use of technology was recognized as one of the primary risk factors negatively impacting not only healthy interpersonal development but also the construction of coherent identities. Expectations and ideals can be a source of stress and anxiety in children and adolescents, as can dysfunctionality and poor familial relationships, experiences of mistreatment, harassment and discrimination, and experiencing poverty and/or humanitarian crises, among others [4, 5].

Finally, the findings of this study reveal the existence of differences in the magnitude and types of emotional or behavioral problems between students belonging to municipal schools and those studying at subsidized or private institutions. Children and adolescents who study in municipal schools have higher scores in the report of contextual conflicts with the family where they perceive tension, communication problems, misunderstanding, loneliness or rejection. Likewise, lower scores are observed in the report of personal resources associated with integration and social competence, where students tend to evaluate negatively their own capacity or ability to initiate and maintain friendship relationships or integrate into groups.

These results reveal the difficulties faced by children and adolescents from vulnerable families, where the lack of resources makes it difficult for parents to adequately raise their children and meet affection, learning, protection or health needs, since they must play the role of provider and work several hours a day neglecting their parenting responsibilities; As a result, their children may perceive an absence of sources of support and this experience may mean that their parents have become helpless or abandoned and that they have become disengaged from their own family [40–42]. Some studies indicate that families with fewer resources are those who face more domestic violence due to setbacks or economic crises, exposing their children to experiences of victimization or poly-victimization that compromise an endemic state of defenselessness where the child or adolescent assumes as permanently probable the aggression or harm of others [43, 44]. The latter may explain the finding of this study related to the difficulties students report in feeling able to relate to others or integrate into groups, since experiences of neglect, abandonment, mistreatment or abuse in their family trigger fears that condition their interpersonal development, tending to adopt a defensive attitude that leads them to isolate themselves in order to reduce the likelihood of harm from their peers [45].

On the other hand, students who belong to private schools tend to present higher scores in internalizing problems during the period of Secondary School associated with both depression, where there are feelings of sadness, frustration, irritability, constant tiredness or fatigue, difficulties in focusing attention, remembering or understanding in addition to isolation or withdrawal, as well as social anxiety whose greatest fear is negative evaluation or rejection by their own peers. This last finding is consistent with the high scores registered in contextual problems related to their own peers where students perceive being constantly rejected or isolated by their peers, the same scenario that is observed in students from subsidized schools. Finally, low scores are observed in relation to the personal resource of self-esteem revealing in students dissatisfaction with themselves and negative evaluations of their self-concept.

The findings described show the difficulties that adolescents enrolled in private or subsidized educational establishments tend to face, whose interpersonal problems and the presence of social anxiety can be explained by the need for acceptance and group membership, For even when they are studying in private schools, their peers tend to make social distinctions that reveal economic inequalities and promote segregation or discrimination, presenting those students who are segregated or discriminated against with feelings of inadequacy and maladjustment, as well as fears and a strong appreciation of the criticism or opinion of others [46]. On the other hand, it has been estimated that families with greater economic resources tend to have high expectations about the educational development and growth of their children, which makes the presence of demands, pressures, criticism, comparisons, disapproval and the absence of recognition of the school performance they observe in them probable; those who tend to present symptoms of depression, such as insecurities, excessive self-criticism, guilt, stress, feelings of inferiority or frustration, low self-esteem and lack of motivation [47].

It is important to mention that Chile is the ninth most unequal OECD country in the world, where the 10% of the population with the most resources has an income 27 times higher than the 10% most vulnerable in the country, as a result of public policies that allow for a high degree of privatization of basic services, observing important socio-economic gaps that impact the quality of life, these being more visible in the public and private provision of health services, housing, work, security and education [48].

With regard to the differences between private and public education, it has been noted that the country’s most vulnerable student population tends to begin and complete their studies in public educational establishments, whose quality is not comparable to that received by students enrolled in private schools, given the differences in the investment of resources for stimulating learning. Consequently, the socio-economic capacity of families tends to determine the quality of education that their children will receive, in addition to the learning challenges and the magnitude or types of psychosocial problems that they must address [49].

Chile belongs to the 10% of nations worldwide that do not constitutionally recognize education as a universal right, empowering the market to arbitrate the distribution of resources and the quality of educational training for Chilean children and adolescents according to their social and economic realities, delegating to the most vulnerable families the responsibility of providing quality learning to their children, given that public educational establishments do not have sufficient and necessary resources to do so, promoting profound inequalities that in the long term impede social mobility and determine as permanently probable the condition of vulnerability in families and the low expectations of educational growth or development in their children [50].

Among the main limitations of this study is the fact that the students’ reading comprehension levels turned out to be a difficulty for the execution of practical indications and instructions regarding the questionnaires. Further, the resistance of educational institutions to collaborate with the study precluded the attainment of a larger sample.

It is important to consider that this research used only self-reports as a means of collecting information regarding students’ emotional and behavioral problems, as well as other studies aimed at exploring or analyzing psychological problems in childhood or adolescence and their relationship with family climate, attachment, stress from traumatic experiences and learning difficulties, among others [51–53], this being a limitation that makes it impossible to know the impressions of parents or teachers about the mental health of their children or students. Consequently, this study cannot compare which characteristics, dynamics or modalities should be considered in an intervention or training plan to address mental health problems in children and adolescents that includes both actions in educational and family contexts.

Future studies should consider the design and evaluation of psychosocial interventions aimed at strengthening emotional regulation and self-esteem in school settings, along with exploring and analyzing potential differences between subgroups, especially the most vulnerable (such as immigrants, indigenous peoples, and all those within each educational establishment who present some type of symptomatology), in an attempt to ensure plans of action are available to assist in the care of these children and adolescents.

## Conclusion

The findings of this study showed that the main mental health problems of children and adolescents in northern Chile are anxiety, depression, and behavioral disorders. Differences by sex were observed, as the female population is the most affected, and there is a high probability that these problems will extend into adulthood. In addition, areas of vulnerability included students’ difficulties with regulating their emotions, and, in adolescents, the search for sensations and the risk factors of low self-esteem, difficulty in becoming aware of problems, and low social competence. The results suggested the importance of timely intervention for the diagnosis and treatment of children and adolescents with their families, with the aim of reducing the likelihood these psychiatric disorders will persist and even worsen in adulthood.

## Data Availability

The datasets generated and/or analysed during the current study are not publicly available due Government policy but are available from the corresponding author on reasonable request.

## Abbreviations

ADHD: Attention Deficit Hyperactivity Disorder
DPD: Dissocial Personality Disorder
ODD: Oppositional Defiant Disorder
*SENA*: *Sistema de Evaluación de Niños y Adolescentes*
